# Seroprevalence of Measles-, Mumps- and Rubella-Specific IgG Antibodies in German Children and Adolescents and Predictors for Seronegativity

**DOI:** 10.1371/journal.pone.0042867

**Published:** 2012-08-06

**Authors:** Christina Poethko-Müller, Annette Mankertz

**Affiliations:** 1 Department of Epidemiology and Health Reporting, Robert Koch Institute, Berlin, Germany; 2 Division of Viral Infections; National Reference Center MMR and RRL WHO EURO, Robert Koch Institute, Berlin, Germany; Health Protection Agency, United Kingdom

## Abstract

We have undertaken a seroprevalence study with more than 13,000 children, who had been included in the German KIGGS survey, a representative sample of children and adolescents 0–17 years of age. The IgG titres against measles, mumps and rubella were determined in 1 to 17 year olds While 88.8% of the children were MMR-vaccinated at least once, 76.8% of children aged 1 to 17 years showed prevalence of antibodies to MMR. The highest seronegativity was seen with respect to mumps. Gender differences were most pronounced with regard to rubella IgG titres: girls aged 14 to 17 years were best protected, although seronegativity in 6.8% of this vulnerable group still shows the need of improvement. Search for predictors of missing seroprevalence identified young age to be the most important predictor. Children living in the former West and children born outside of Germany had a higher risk of lacking protection against measles and rubella, while children with a migration background but born in Germany were less often seronegative to measles antibodies than their German contemporaries. An association of seronegativity and early vaccination was seen for measles but not for mumps and rubella. A high maternal educational level was associated with seronegativity to measles and rubella. In vaccinated children, seronegativity was highest for mumps and lowest for rubella. For mumps, high differences were observed for seronegativity after one-dose and two-dose vaccination, respectively. Seronegativity increases as time since last vaccination passes thus indicating significant waning effects for all three components of MMR.

## Introduction

Measles is a highly contagious viral disease. In the prevaccination period, >90% of individuals had contracted the disease by the age of 10 years. Typical symptoms are high fever, cough, coryza, conjunctivitis and maculopapular rash. Relatively common complications of measles include otitis media, laryngo-tracheobronchitis and pneumonia. Post-infectious measles encephalitis occurs in about 1/1,000 cases, and subacute sclerosing panencephalitis, a slowly progressing infection of the central nervous system, occurs in about 1/10,000–100,000 cases [1], [2]. Twofold vaccination with a measles containing vaccine (MCV) has substantially reduced the worldwide incidence of measles, although insufficient vaccination coverage has resulted in a resurgence e.g. in several countries of Western Europe [3], among them Germany [4]. The German incidence increased to almost 2 cases/100,000 total population in 2011 with the highest incidence in children still unvaccinated due to young age. Rising incidence rates were also observed for adolescents and young adults [5].

Mumps is a viral infection characterized by parotitis and fever. Although mumps is most frequently reported in children aged 5–9 years, both adolescents and adults may be affected, among whom complications such as meningitis and orchitis are relatively more common. Natural infection is thought to confer lifelong protection. Studies in several countries before the implementation of large-scale childhood vaccination demonstrated that the seroprevalence of antibodies to mumps virus reached approximately 90% in individuals aged 14–15 years [6].

Rubella is an acute, usually mild viral disease. Its public health importance is caused by the teratogenic potential of the virus which may result in miscarriage, fetal death or congenital defects called congenital rubella syndrome (CRS) [7], [8]. The primary goal of rubella vaccination is to prevent congenital rubella infection. Safe and efficacious live-attenuated vaccines are in use to prevent infection with measles, mumps and rubella. Elimination goals have been adopted for several WHO regions. The European Region has established goals to eliminate measles and CRS by 2015 [9].

The German history of measles-, mumps- and rubella vaccinations differed by the former ‘East’ and ‘West’ until 1991. While in the former East Germany mandatory vaccination against measles was in place since 1970 and was augmented by a second measles dose in 1986, mumps and rubella vaccination was not introduced in the former East Germany. Study participants were born between 1985 and 2004. Older children and adolescents who were born in the former East Germany received mumps and rubella vaccinations after the reunification 1990. In the former West Germany, measles vaccination was recommended in 1973 followed by the recommendation of measles-mumps-vaccination in 1976. Rubella vaccination was only recommended for adolescent girls until 1980. In 1980, vaccination against measles, mumps and rubella (MMR) was brought forward to the second year of life for all children; female adolescents should receive rubella vaccination irrespective of an early childhood dose. Recommendation of a general second MMR dose at the age of 5 was released in 1991, and was changed to the second year of life in 2004. Today, a two-dose regime is recommended for MMR by the German Standing Committee of Vaccination (STIKO). The first dose should be given at months 11–14, the second dose not less than 4 weeks later. MMR immunisation should be completed no later than at the age of 2. From 2010 on, measles vaccination recommendation include young adults who are unvaccinated or who had received only one dose of measles vaccine.

Vaccinations with MMR within the first years of life may induce a shift of the incidence to older age groups. Realization of these potential effects may be delayed in Germany as mandatory nation-wide case reporting for mumps and rubella have not been implemented until 2012. Therefore, sero-epidemiological surveys are highly important for the assessment of current gaps in seroprevalence of disease-specific antibodies and possible adjustment of national vaccination policies. Prior to the KiGGS study, no representative sero-epidemiological data on the seroprevalence of measles-, mumps- and rubella antibodies in children existed for Germany. First results of isolated results on measles seroprevalence have been described earlier [10].It is the objective of this study, to describe MMR seroprevalence as a whole and calculate independent predictors for seronegativity for measles-, mumps- and rubella antibodies. The remarkable added value of this study is the combination of seroprevalence results and detailed data on the vaccination status on an individual level.

## Methods

### Survey design and study population

The KiGGS methodology has been described elsewhere [11], [12]. In brief, the KiGGS survey is based on a representative national sample of children and adolescents 0–17 years of age with main residence in Germany. A total of 17,641 children and adolescents were surveyed. Study participants were enrolled from May 2003 to May 2006. The overall response for eligible children and adolescents was 66.6% and showed little variation between age groups and sexes, but marked variation between children with and without migration background. Analyses of the short non-responder questionnaires revealed that the collected data give comprehensive and nationally representative evidence on the health status of children and adolescents aged 0 to 17 years.

Questionnaires for children and a parent collected data on medical history, socioeconomic status and migration background. Data on vaccination was taken directly from the vaccination cards. The age categories correspond to different phases of life for which intensity and pattern health care utilisation is different. The assigned maternal education levels relate to the German school system which provides three different types of secondary education. A high education level was defined for a secondary school leaving certificate graduating for a general university entrance or the entrance to an University of Applied Sciences (Abitur/Fachabitur). A medium level was defined for graduation from an Intermediate Secondary School (‘Realschule’, usually finished after the 10^th^ grade) and a low education level was assigned if a Secondary General School (‘Hauptschule’, usually finished after the 9^th^ grade) had been attended or no formal school graduation had been completed.

In children aged 1 to 17 years, parents and children were asked to consent to taking of a blood sample. Written consent was obtained from parents and from children above the age of 13. In 13,977 (83.7%) study subjects, a blood sample could be taken and subsequently tested for the presence of measles IgG antibodies, 13,930 study subjects were tested for mumps- and 13,968 for rubella IgG antibodies. Presented seroprevalence estimates are based on these groups (paragraph 3.1. and 3.2.). In 93.1% of children who were tested for MMR antibodies, information about vaccinations could be obtained from vaccination cards or parents reported that the children were unvaccinated. Participants with missing or incomplete information on vaccinations were excluded from further uni- and multivariate analyses of determinants of seronegativity (paragraph 3.3. and 3.4.).

### Statistical Analysis

Estimates of vaccination coverage and their confidence intervals (CIs) were calculated using SPSS version 18 (SPSS Inc. Chicago, Illinois). In order to assure that estimates derived from the KiGGS study are representative at the national level, survey weights were applied throughout the statistical analyses. Analyses were performed using SPSS Complex Samples procedure and, thus, accounted for the stratified and clustered sample design of our survey. Calculations of the MMR seroprevalence (paragraph 3.1) and descriptive, uni- and multivariate analyses of measles-, mumps and rubella specific antibodies (paragraph 3.2) stratified by socio-demographic factors (sex, age, migration background, maternal education level) included all children with known titres, regardless of the quality of their vaccination documentation. Dichotomizations were performed by combining positive and equivocal titres in the positive category. A p-value <0.05 was considered to be statistically significant.

**Figure 1:**
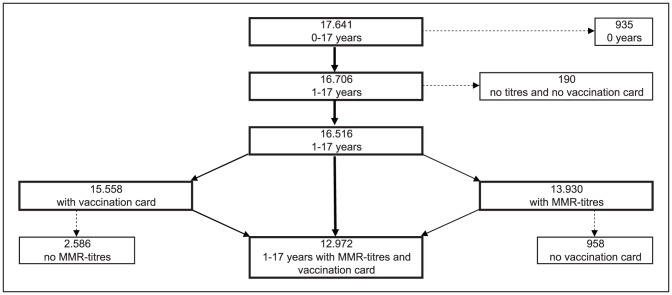
Flow chart on study subjects.

In a second step, the seroprevalence of measles-, mumps and rubella specific antibodies was stratified by factors related to vaccination status (number of vaccination doses, age at first vaccination, years since last vaccination, history of the respective infection). In these analyses only vaccinated children were included for whom a valid vaccination card was presented (Figure 1). Children whose blood sample was taken within 21 days after their first vaccination (measles: n = 30; mumps: n = 32; rubella: n = 36) were excluded from the analyses (paragraph 3.3 and 3.4). The MMR-vaccination rate (paragraph 3.1) was obtained in a subset of 12,972 children for whom a vaccination card was provided or for whom parents reported that they were (yet) unvaccinated.

### Laboratory methods

#### Measles IgG ELISA

The measles IgG titre of all serum samples was determined by the Siemens Enzygnost anti-measles IgG test (Siemens, Marburg, Germany) using an automated processor (Tecan Evolyzer, Crailsheim, Germany). All samples were tested with kits of the same lot number. The result of the ELISA was expressed quantitatively as an antibody concentration (mIU/ml) of optical density (OD) according to the manufacturer's instructions. Samples were categorised as seropositive, equivocal or seronegative according to the cut-off values proposed by the manufacturer. Based on the widely agreed categories for IgG antibody negativity (IgG titre <150 mIU/ml), seropositivity (IgG titre >350 mIU/ml) or equivocal measles antibody levels (IgG titre 150–350 mIU/ml), the obtained OD/IgG titre was categorised taking into account the respective manufacturers correction factors. Internationally, the value of 0.2 IU/ml is seen as a correlate of protection. Thus, the group of children with equivocal titres can be assumed to be protected against measles.

#### Mumps IgG ELISA

The mumps IgG titre of all serum samples was determined by the Euroimmune anti-mumps-virus-AT-ELISA (IgG) (Euroimmun, Luebeck, Germany) using an automated processor (Tecan Evolyzer, Crailsheim, Germany). All samples were tested with kits of the same lot number. The result of the ELISA was calculated by correlation to a standard curve and expressed in relative units (RU)/ml. This result was interpreted according to the manufacturer's recommendation as negative for titres <16 RU/ml, equivocal for titres ≥16 and <22 RU/ml and positive for titres ≥22 RU/ml.

#### Rubella IgG ELISA

The rubella IgG titre of all serum samples was determined by the Euroimmune anti-rubella-virus-ELISA (IgG) (Euroimmun, Luebeck, Germany) using an automated processor (Tecan Evolyzer, Crailsheim, Germany). All samples were tested with kits of the same lot number. The result of the ELISA was calculated by correlation to a standard curve and expressed in international units (IU)/ml. This result was interpreted according to the manufacturer's recommendation as negative for titres <8 IU/ml, equivocal for titres ≥8 and <11 IU/ml and positive for titres ≥11 IU/ml.

### Ethics Statement

The study was approved by the Charité-Universitätsmedizin Berlin ethics committee and the Federal Office for the Protection of Data.

## Results

### Seroprevalence of MMR antibodies

The overall distribution of antibodies to measles, mumps and rubella in children tested for MMR IgG antibodies is shown in table 1. The prevalence of IgG antibodies to MMR in children aged 1–17 years was, on average, 76.8% (95% CI 75.7–78.0).

**Table 1:** Seroprevalence of MMR IgG-Titres and MMR vaccination rate (at least one dose) by age.

	Age [years]					
	1–2	3–6	7–10	11–13	14–17	Total
**IgG antibody combination**						
MMR positive	67.2% (63.8–70.5)	81.3% (79.2–83.3)	80.2% (78.4–81.8)	75.1% (72.9–77.1)	75.2% (73.3–77.0)	76.8% (75.7–78.0)
Measles and rubella positive	6.6% (5.0–8.6)	6.8% (5.6–8.2)	7.3% (6.2–8.6)	8.8% (7.5–10.3)	8.7% (7.5–10.3)	7.8% (7.0–8.7)
Measles and mumps positive	0.2% (0.1–0.5)	2.2% (1.5–3.3)	3.1% (2.3–4.1)	5.3% (4.2–6.7)	5.1% (4.1–6.3)	3.6% (3.0–4.4)
Rubella and mumps positive	3.4% (2.3–4.9)	2.2% (1.6–3.0)	2.3% (1.8–2.9)	3.6% (2.9–4.5)	4.1% (3.4–4.9)	3.1% (2.7–3.5)
Measles positive	0.2% (0.0–0.6)	1.1% (0.7–1.7)	1.9% (1.5–2.5)	2.3% (1.7–3.0)	2.4% (1.9–3.0)	1.8% (1.5–2.0)
Rubella positive	0.8% (0.3–2.0)	0.5% (0.3–1.0)	0.9% (0.6–1.3)	1.5% (1.1–2.1)	2.2% (1.7–2.8)	1.3% (1.1–1.5)
Mumps positive	2.8% (1.8–4.4)	0.9% (0.5–1.4)	1.2% (0.8–1.7)	0.9% (0.6–1.4)	0.8% (0.5–1.1)	1.1% (0.9–1.4)
MMR negative	18.8% (16.2–21.8)	5.0% (4.1–6.0)	3.2% (2.6–3.9)	2.5% (1.9–3.3)	1.6% (1.1–2.1)	4.5% (4.1–4.9)
**MMR-vaccination rate**	78.9% (76.0–81.6)	92.4% (90.8–93.7)	92.5% (91.1–93.7)	89.4% (87.6–91.0)	85.8% (83.9–87.6)	88.8% (87.6–89.8)

Distribution of antibodies to measles, mumps and rubella in German children aged 1–17 year; vaccination coverage of at least one dose of measles, mumps and rubella vaccine (mono-, bi- or three-valent vaccines) in those who provided their vaccination card.

Prevalence of MMR antibodies was highest in 3–6 year old children (81.3; 95% CI 79.2–83.3). MMR antibody prevalence was lowest in 1–2 year old children (67.2; 95% CI 63.8–70.5) but was also low in 11–13 and 14–17 year old children and adolescents (75.1%; 95% CI 72.9–77.1 and 75.2%; 95% CI 73.3–77.0, respectively). The prevalence of antibodies to measles and rubella in the absence of mumps specific antibodies was the second most frequent pattern of antibody prevalence (7.8%; 95% CI 7.0–8.7).

The MMR-vaccination rate corresponds best to the prevalence of MMR antibodies in 1–6 year old children while the vaccination rate of at least one MMR vaccination was considerable higher than the concomitant prevalence of all three IgG antibody types in older children. Discrepancies were highest for mumps-specific antibodies.

**Figure 2:**
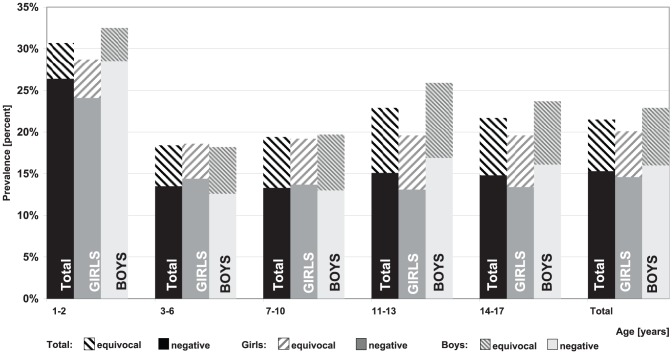
Prevalence of negative and equivocal Mumps IgG antibody titres by age and gender.

**Figure 3:**
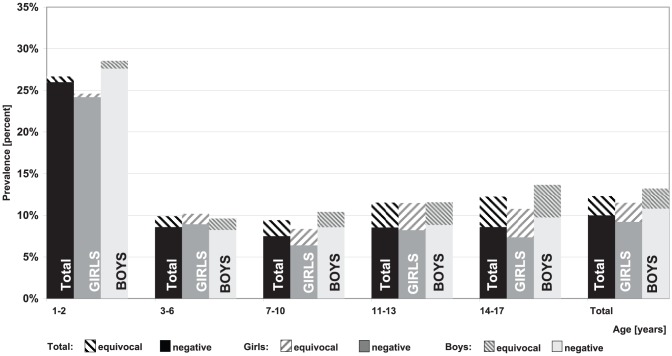
Prevalence of negative and equivocal Measles IgG antibody titres by age and gender.

**Figure 4:**
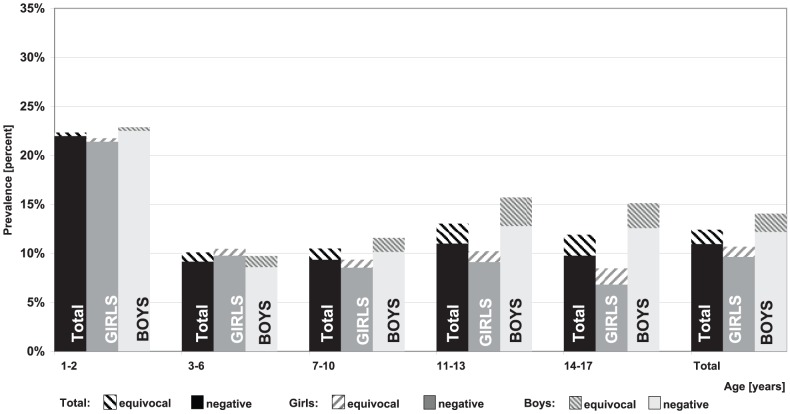
Prevalence of negative and equivocal Rubella IgG antibody titres by age and gender.

Overall, the highest level of seronegativity was seen with regard to mumps specific antibodies (15.3%; 95% CI 14.4–16.3) (Figure 2) and also the prevalence of children with equivocal titre level was higher for mumps antibodies than for measles (Figure 3) and rubella (Figure 4) antibodies. Some differences for gender and age were seen: In general, girls had a lower rate of seronegativity although this pattern was not seen in 3–6 year olds. The effect was more pronounced for rubella titres, especially in 11–17 year old boys. In 11–13 year old girls, rubella seronegativity of was, on average, 9.1% (95% CI 7.4–11.3) whereas in boys the proportion was 12.8% (95% CI 10.7–15.2). Differences were even more pronounced between 14–17 year old girls and boys with 6.8% (95% CI 5.3–8.7) and 12.6% (95% CI 10.8–14.6), respectively.

### Seroprevalence of measles-, mumps and rubella antibodies and predictors of missing seroprevalence

A detailed overview on population-based positive, negative and equivocal antibody titres to measles-, mumps and rubella in all children tested for antibodies is given in table 2.

**Table 2:** Seroprevalence of measles-, mumps- and rubella lgG antibody titres in German children by sociodemografic factors.

	Measles Titre			Mumps Titre			Rubella Titre		
	Negative %(95% CI)	Borderline %(95% CI)	Positive %(95% CI)	Negative %(95% CI)	Borderline %(95% CI)	Positive %(95% CI)	Negative %(95% CI)	Borderline %(95% CI)	Positive %(95% CI)
**Total**	10.0 (9.4–10.7)	2.3 (2.0–2.7)	88.5 (87.6–89.3)	15.3 (14.4–16.3)	6.2 (5.8–6.7)	78.4 (77.4–79.4)	11.0 (10.0–11.9)	1.5 (1.3–1.7)	87.6 (86.7–88.5)
**Gender**									
Male	10.8 (9.9–11.6)	2.4 (2.0–2.9)	86.9 (85.9–87.9)	16.0 (14.9–17.2)	6.9 (6.9–7.6)	77.1 (75.8–78.3)	12.2 (11.2–13.3)	1.9 (1.5–2.2)	86.0 (84.9–87.0)
Female	9.2 (8.5–10.0)	2.3 (1.9–2.8)	88.5 (87.6–89.3)	14.6 (13.5–15.8)	5.5 (4.9–6.2)	79.9 (78.4–81.2)	9.6 (8.5–10.9)	1.0 (0.8–1.3)	89.3 (88.1–90.4)
**Age (years)**									
1–2	26.0 (23.2–29.0)	0.7 (0.3–1.6)	73.3 (70.2–76.2)	26.4 (23.3–29.7)	4.3 (3.0–6.2)	69.3 (65.8–72.6)	22.0 (19.3–24.9)	0.4 (0.1–1.3)	77.7 (74.7–80.4)
3–6	8.6 (7.4–9.9)	1.3 (0.9–1.9)	90.1 (88.6–91.4)	13.5 (11.9–15.1)	4.9 (4.1–6.0)	82.2 (80.3–84.0)	9.2 (7.7–10.9)	0.9 (0.6–1.4)	89.9 (88.2–91.4)
7–10	7.5 (6.5–8.6)	1.9 (1.4–2.5)	90.6 (89.3–91.7)	13.3 (11.9–14.8)	6.1 (5.2–7.2)	80.6 (78.7–82.3)	9.4 (8.1–10.8)	1.1 (0.8–1.6)	89.5 (88.1–90.7)
11–13	8.5 (7.5–9.7)	3.0 (2.3–3.9)	88.5 (87.2–89.7)	15.1 (13.4–16.9)	7.8 (6.7–9.0)	77.2 (75.1–79.1)	11.0 (9.5–12.7)	2.0 (1.5–2.7)	87.0 (85.4–88.4)
14–17	8.6 (7.5–9.8)	3.7 (2.9–4.5)	87.8 (86.3–89.0)	14.8 (13.3–16.4)	6.9 (6.1–7.9)	78.2 (76.3–80.0)	9.8 (8.4–11.3)	2.1 (1.7–2.7)	88.1 (86.6–89.5)
**Place of residence**									
Former East	8.1 (7.2–9.0)	2.8 (2.2–3.6)	89.1 (88.0–90.1)	14.8 (11.6–18.7)	5.5 (4.7–6.5)	79.5 (75.5–82.9)	7.8 (6.9–8.9)	1.6 (1.1–2.3)	90.5 (89.4–89.4)
Former West	10.4 (9.7–11.1)	2.3 (1.9–2.7)	87.3 (86.5–88.2)	15.5 (14.6–16.3)	6.3 (5.8–6.9)	78.2 (77.2–79.2)	11.6 (10.5–12.7)	1.4 (1.2–1.7)	87.0 (85.9–88.0)
**Migration background**									
German-born	8.5 (7.2–9.9)	1.0 (0.7–1.6)	90.5 (89.0–91.8)	16.0 (14.3–17.8)	5.8 (4.8–6.9)	78.2 (76.3–80.1)	10.0 (8.6–11.6)	1.4 (0.9–2.0)	88.6 (87.0–90.1)
Foreign-born	14.7 (11.7–18.4)	8.4 (6.3–11.1)	76.9 (73.0–80.3)	18.7 (15.2–22.7)	5.9 (4.2–8.2)	75.5 (71.0–79.4)	19.5 (16.3–23.1)	0.9 (0.4–2.2)	79.6 (75.8–82.9)
None	10.0 (9.3–10.7)	2.3 (2.0–2.7)	87.7 (86.9–88.5)	14.8 (13.9–15.9)	6.4 (5.8–6.9)	78.8 (77.6–79.9)	10.5 (9.6–11.6)	1.5 (1.3–1.8)	88.0 (87.0–88.9)
**Maternal education level**									
High	11.9 (10.8–13.2)	2.4 (1.9–3.1)	85.6 (84.2–86.9)	16.1 (14.9–17.5)	5.9 (5.1–6.8)	77.9 (76.6–79.3)	13.8 (12.2–15.5)	1.3 (1.0–1.8)	84.9 (83.2–86.4)
Medium	9.7 (8.8–10.7)	2.4 (2.0–2.9)	87.9 (86.8–88.9)	14.1 (12.9–15.4)	6.5 (5.9–7.3)	79.3 (77.8–80.8)	9.8 (8.7–11.0)	1.4 (1.1–1.8)	88.8 (87.7–89.9)
Low	8.5 (7.5–9.6)	2.1 (1.6–2.7)	89.5 (88.3–90.6)	16.0 (14.4–17.7)	5.9 (5.1–6.8)	78.1 (76.2–79.9)	9.4 (8.1–10.9)	1.8 (1.3–2.3)	88.8 (87.3–90.2)
**History of respective infection**									
No	10.3 (9.6–11.0)	2.4 (2.0–2.8)	87.4 (86.5–88.1)	15.2 (14.3–16.1)	6.1 (5.6–6.6)	78.7 (77.7–79.7)	10.7 (9.8–11.8)	1.5 (1.3–1.7)	87.8 (87.2–89.2)
Yes	8.6% (6.7–10.9)	1.9 (1.1–3.2)	89.5 (86.8–91.7)	12.4 (9.7–15.8)	6.2 (4.3–8.9)	81.4 (76.9–85.2)	10.9 (9.1–13.1)	1.8 (1.1–2.8)	87.3 (85.1–89.3)
Don't know	7.5 (5.3–10.6)	2.4(1.3–4.3)	90.1 (86.6–92.7)	14.5 (11.4–18.3)	8.4 (5.6–12.3)	77.1 (72.0–81.5)	12.3 (9.9–15.1)	2.3 (1.3–3.9)	85.5 (82.4–88.1)

Generally, seronegativity was highest in one and two year old children. Differences by gender were most obvious with regard to rubella; seronegativity was higher in male than in female children. Seronegativity to rubella was also higher in children living in the former West Germany; differences were smaller for measles and no difference by place of residence was seen with regard to mumps antibodies.

Seronegativity to measles and rubella was higher in children who were foreign-born than in children who were German-born or had no migration background whereas no difference was seen with regard to mumps antibodies. The difference was most pronounced for the category of equivocal measles antibody titres between foreign-born and German-born children with very high equivocal titres in children who were foreign-born. Seronegativity to measles and rubella antibodies was higher if the maternal education level was high while no difference was seen with regard to mumps antibodies.

In order to identify and compare factors predicting the odds of negative measles-, mumps and rubella antibody titres, we performed detailed population-based multivariate analyses for each of the three different antibodies (Table 3).

**Table 3:** Multivariate odds ratios (OR) for the association between sociodemographic factors and the risk of a negative measles-, mumps- and rubella antibody titres in German children and adolescents.

	Measles		Mumps		Rubella	
	multivariate OR* (95% CI)	p value	multivariate OR** (95% CI)	p value0.037	multivariate OR*** (95% CI)	p value
**Gender**		0.008				<0.001
male	1.18 (1.04–1.32)		1.13 (1.01–1.27)		1.29 (1.12–1.48)	
female	Referent		Referent		Referent	
**age (years)**		<0.001		<0.001		<0.001
1–2	3.69 (2.94–4.63)		2.10 (1.68–2.62)		2.60 (1.98–3.41)	
3–6	1.02 (0.82–1.26)		0.91 (0.76–1.09)		0.98 (0.78–1.24)	
7–10	0.87 (0.71–1.07)		0.89 (0.74–1.07)		0.99 (0.81–1.20)	
11–13	0.95 (0.79–1.16)		1.00 (0.83–1.21)		1.14 (0.94–1.38)	
14–17	Referent		Referent		Referent	
**migration background**		<0.001		0.152		<0.001
German-born	0.77 (0.64–0.94)		1.03 (0.88–1.20)		0.88 (0.72–1.06)	
Foreign-born	1.89 (1.40–2.56)		1.34 (0.99–1.81)		2.19 (1.71–2.82)	
None	Referent		Referent		Referent	
**Maternal education level**		0.003		0.055		<0.001
High	1.37 (1.14–1.64)		0.97 (0.82–1.41)		1.51 (1.25–1.82)	
Medium	1.18 (0.98–1.42)		0.86 (0.74–1.00)		1.10 (0.91–1.33)	
Low	Referent		Referent		Referent	
**Place of residence**		<0.001		0.724		<0.001
Former East	Referent		Referent		Referent	
Former West	1.43 (1.24–1.66)		1.05 (0.79–1.41)		1.57 (1.28–1.92)	

Legend Table 3: All multivariate models were adjusted for the parental reported history of the respective (measles/mumps/rubella) infection.

* explained variation [Nagelkerke]: 0.056; n = 13.091.

** explained variation [Nagelkerke]: 0.019; n = 13.044.

*** explained variation [Nagelkerke]: 0.040; n = 13.082;

Children aged 1 and 2 years had the highest odds of being seronegative. The comparison of results between measles-, mumps- and rubella specific IgG antibodies showed that the association between seronegativity and young age was strongest for measles. The odds of being seronegative for measles was 3.69 (95% CI 2.94–4.63) in children aged 1–2 years compared to adolescents aged 14–17 years (mumps: OR 2.1; 95% CI 1.68–2.62; rubella: OR 2.60; 95% CI 1.98–3.41).

The odds of being seronegative were also high with regard to measles- and rubella specific antibodies for children who were foreign-born (OR 1.89; 95% CI 1.40–2.56 and OR 2.19; 95% CI 1.71–2.82, respectively). Children with migration background who were born in Germany had a lower odds of being seronegative for measles. No such association was observed for mumps.

Boys were more likely to be seronegative to measles, mumps and rubella than girls; the difference was strongest for rubella. Children living in the region of the former West Germany were more likely to be seronegative to measles and rubella than children in the former East Germany. The odds of being seronegative was 1.43 (95% CI 1.24–1.66) for measles and was 1.57 (95% CI 1.28–1.91) for rubella; no difference by place of residence was seen for mumps antibodies.

A high maternal education level was associated with seronegativity to measles and rubella. The odds of being seronegative for measles was 1.37 (95% CI 1.14–1.64) for children whose maternal education level was high (reference group: low maternal education level) and was 1.51 (95% CI 1.25–1.82) for seronegativity to rubella.

Interaction between age and gender was not shown for seronegativity to measles but was significant for seronegativity to mumps and rubella. Stratified analyses showed that the higher odds of being seronegative for mumps- and rubella in boys was restricted to the group of children aged 11–17 years.

### Seroprevalence of MMR antibodies and vaccination status related factors

A detailed overview on positive, negative and equivocal antibody titres to measles-, mumps and rubella in children for whom parents provided a vaccination card or reported that they were (yet) unvaccinated is given in table 4. Seronegativity to measles, mumps and rubella was higher in children without a vaccination card than in children with a valid vaccination card.

**Table 4:** Seroprevalence of measles-, mumps- and rubella lgG antibody titres in children by documentation of vaccinations and by vaccination status (children with documentation of vaccinations).

	Measles Titre			Mumps Titre			Rubella Titre		
	Negative %(95% CI)	Borderline %(95% CI)	Positive %(95% CI)	Negative %(95% CI)	Borderline %(95% CI)	Positive %(95% CI)	Negative %(95% CI)	Borderline %(95% CI)	Positive %(95% CI)
**Total (no vaccination card)**	16.0 (13.4–19.1)	3.3 (2.3–4.7)	80.7 (77.5–83.5)	24.3 (21.0–27.9)	5.4 (4.2–7.1)	70.3 (66.7–73.6)	18.4 (15.8–21.3)	1.4 (0.8–2.3)	80.2 (77.3–82.8)
**Total (with vaccintion card)**	9.5 (8,9–10.2)	2.3 (2.0–2.6)	88.2 (87.5–88.9)	14.6 (13.7–15.6)	6.3 (5.8–6.8)	79.1 (78.0–80.1)	10.4 (9.5–11.3)	1.5 (1.3–1.7)	88.2 (87.3–89.1)
**Vaccination status**									
Unvaccinated	68.8 (65.2–72.2)	0.8 (0.4–1.6)	30.4 (27.1–34.0)	65.9 (62.5–69.2)	5.2 (3.7–7.2)	28.9 (25.8–32.2)	68.3 (64.4–71.9)	0.6 (0.3–1.4)	31.1 (27.4–35.0)
Single dose vaccination	6.5 (5.4–7.8)	2.4 (1.7–3.2)	91.1 (89.6–92.5)	19.3 (17.3–21.4)	9.9 (8.9–11.0)	70.8 (58.6–73.0)	5.9 (4.9–7.1)	1.6 (1.2–2.1)	92.5 (91.3–93.6)
Two-dose (or more) vaccination	4.3 (3.8–4.9)	2.4 (2.0–2.8)	93.3 (92.6–93.9)	7.3 (6.4–8.3)	5.3 (4.8–5.8)	87.4 (86.3–88.4)	2.2 (1.6–3.1)	1.6 (1.3–1.9)	96.2 (95.3–96.9)
**Years since last vaccination**									
***One dose***									
Years since last vaccination 0–2	6.7 (4.8–9.1)	1.4 (0.7–2.8)	91.9 (89.0–94.1)	17.6 (14.9–20.7)	8.7 (6.7–11.3)	73.7 (70.0–77.2)	4.2 (3.0–5.8)	0.3 (0.1–0.9)	95.5 (93.9–96.8)
Years since last vaccination 3–6	6.0 (4.0–8.9)	1.4 (0.8–2.7)	92.6 (89.5–94.8)	19.2 (15.7–23.2)	8.9 (7.1–11.1)	72.0 (67.6–75.9)	3.9 (2.5–6.0)	1.0 (0.5–1.8)	95.1 (93.1–96.6)
Years since last vaccination >6	6.9 (5.3–8.9)	3.7 (2.5–5.5)	89.4 (87.0–91.4)	20.6 (18.0–23.5)	11.5 (9.7–13.5)	67.9 (64.7–70.9)	9.7 (7.9–11.9)	3.6 (2.6–5.0)	86.8 (84.4–88.8)
***Two (or more) doses***									
Years since last vaccination 0–2	2.7 (2.1–3.4)	1.4 (1.0–1.8)	96.0 (95.2–96.6)	5.4 (4.4–6.7)	4.0 (3.3–4.8)	90.6 (89.1–91.9)	1.1 (0.6–1.8)	0.4 (0.3–0.7)	98.5 (97.7–99.0)
Years since last vaccination 3–6	4.5 (3.8–5.3)	2.9 (2.3–3.6)	92.6 (91.7–93.5)	8.3 (7.3–9.5)	6.2 (5.4–7.1)	85.5 (84.1–86.8)	3.0 (2.1–4.4)	2.3 (1.8–3.0)	94.7 (93.4–95.8)
Years since last vaccination >6	8.4 (7.0–10.1)	4.1 (3.1–5.5)	87.4 (85.3–89.3)	10.4 (8.3–12.9)	6.9 (5.5–8.6)	82.8 (79.6–85.6)	4.4 (3.2–6.0)	3.6 (2.6–5.1)	92.0 (90.0–93.6)
**Age at first measles vaccination**									
First dose aged 0–11 months	9.6 (7.4–12.3)	3.3 (2.1–5.2)	87.1 (84.3–89.5)	8.5 (5.5–13.0)	6.2 (3.9–9.7)	85.3 (80.4–89.2)	2.2 (1.1–4.5)	0.4 (0.1–1.2)	97.4 (95.0–98.7)
First dose aged 1–17 years	4.5 (4.1–5.1)	2.3 (2.0–2.7)	93.1 (92.5–93.7)	10.2 (9.3–11.2)	6.4 (5.9–6.9)	83.4 (82.3–84.5)	3.3 (2.7–4.1)	1.6 (1.4–1.9)	95.1 (94.3–95.8)
**History of respective infection**									
***Unvaccinated***									
No	81.4 (77.4–84.8)	0,6 (0,2–1,5)	18.0 (14.6–22.0)	72.4 (68.3–76.2)	4.8 (3.2–7.2)	22.8 (19.3–26.7)	75.0 (71.1–78.5)	0.7 (0.3–1.7)	24.3 (20.8–28.3)
Yes	21.3 (14.5–30.1)	0,0	78.7 (69.9–85.5)	15.0 (8.4–25.4)	2.1 (0.5–9.3)	82.8 (71.8–90.1)	40.8 (33.3–48.8)	0.0 (0.0–0.0)	59.2 (51.2–66.7)
Don't know	25.9 (12.4–46.2)	3.5(0.5–21.4)	70.6 (50.2–85.2)	49.5 (30.9–68.3)	8.5 (2.1–28.5)	42.0 (23.4–63.1)	52.4 (39.9–64.7)	1.7 (0.2–10.8)	45.9 (34.5–57.7)
***Vaccinated***									
No	4.8 (4.2–5.4)	2.4 (2.0–2.8)	92.9 (92.1–93.5)	9.9 (9.0–10.8)	6.2 (5.7–6.8)	83.9 (82.9–84.9)	3.1 (2.4–4.0)	1.5 (1.3–1.8)	95.3 (94.5–96.1)
Yes	5.0 (3.5–7.2)	2.4 (1.4–4.2)	92.6 (89.8–94.6)	10.9 (8.2–14.3)	7.8 (5.2–11.7)	81.3 (76.5–85.3)	3.5 (2.3–5.2)	1.9 (1.1–3.3)	94.6 (92.6–96.1)
Don't know	5.0 (3.3–7.6)	2.2 (1.1–4.2)	92.8 (89.3–95.2)	12.2 (8.9–16.6)	8.7 (5.6–13.1)	79.1 (73.3–84.0)	4.8 (3.1–7.3)	2.7 (1.5–4.8)	92.6 (89.7–94.7)

Seroprevalence in children for whom parents provided a vaccination card or reported that they were (yet) unvaccinated was stratified by vaccination related variables. The most important factor associated with seroprevalence of measles, mumps and rubella antibody titres was the respective vaccination; two third of unvaccinated children were seronegative for measles, mumps and rubella antibodies. Seronegativity was lower in children who had received a second dose of the respective vaccine than those who had received a single dose. Seronegativity in one-dose vaccinees was highest with regard to mumps. While 65.9% (95% CI 62.5–69.2) of unvaccinated children were seronegative for mumps antibodies, 19.3% (95% CI 17.3–21.4) were seronegative although they had received one dose of mumps vaccine. Seronegativity to measles and rubella seroprevalence in one-dose vaccinees was 6.5 (95% CI 5.4–7.8) and 5.9% (95% CI 4.9–7.1), respectively. Also seronegativity in two-dose vaccinees was highest with regard to mumps specific antibodies (7.3%; 95% CI 6.4–8.3), and was lowest with regard to rubella (2.2%; 95% CI 1.6–3.1). Seronegativity to measles was 4.3% (95% CI 3.8–4.9) in children who had received two (or more) doses of measles vaccine.

We determined differences by the time passed since last vaccination stratified by the number of vaccine doses. In children who had received a two-dose vaccination, seronegativity was higher in those who had received their last vaccination more than two years before the study in comparison to children who had received their last vaccination no more than two years before. Correspondingly, the proportion of children with equivocal titres was the higher the longer the time period since last vaccination had been. The highest seronegativity accompanied by the highest prevalence of equivocal titres in vaccinated children was shown with regard to mumps in children who had received only one dose of mumps vaccine more than 6 years ago: 20.6% (95% CI 18.0–23.5) were seronegative and 11.5% (95% CI 9.7–13.5) displayed equivocal mumps antibody titres. However, regardless of the number of doses, the relative difference in comparison to children who had received their last dose no more than 2 years ago was lower for mumps than for measles and rubella.

Seronegativity to measles but not to mumps and rubella was higher in children who had received the first vaccination during their first year of life in comparison to children who had received it later.

In unvaccinated children, seronegativity to measles, mumps and rubella was lower in children for whom parents reported the respective history of a natural infection. The predictive value of a parental reported natural infection was lowest for rubella with 40.8% (95% CI 33.3–48.8) children being seronegative although parents reported the history of a rubella infection (measles: 21.3%; 95% CI 14.5–30.1 and mumps: 15.0%; 95% CI 8.4–25.4). No differences in antibody prevalence by parental reported history of a natural infection were seen in vaccinated children.

### Risk of missing seroprevalence by time period since last vaccination

Odds of being seronegative in spite of documented vaccination was investigated in the respective subgroups of vaccinated children for whom the date of the last measles-, mumps- and rubella vaccination was documented in the vaccination card and the respective (measles-, mumps- and rubella) vaccination had been received more than 21 days before blood samples had been taken. Analyses were adjusted for variables that proved out to be significant in the univariate analyses and for the parental report of the respective (measles/mumps/rubella) infection (Table 5).

**Table 5:** Multivariate odds ratios (OR) for the association between time since last vaccination and the risk of a negative measles-, mumps- and rubella antibody titres in German children and adolescents.

	Measles		Mumps		Rubella	
	multivariate OR* (95% CI)	p value	multivariate OR** (95% CI)	p value	multivariate OR*** (95% CI)	p value
**Time since last vaccination**		<0.001		<0.001		<0.001
*One dose*						
0–2 years	1.46 (0.91–2.35)		3.73 (2.61–5.31)		2.11 (1.03–4.34)	
3–4 years	3.47 (1.79–6.71)		4.02 (2.64–6.12)		3.69 (1.70–7.99)	
5–6 years	2.39 (1.25–4.57)		3.86 (2.50–5.97)		4.25 (1.73–10.43)	
7–8 years	3.44 (1.82–6.52)		4.67 (3.23–6.76)		8.63 (3.50–21.27)	
>8 years	2.69 (1.67–4.35)		3.37 (2.47–4.59)		12.79 (5.58–29.30)	
*Two (or more) doses*						
0–2 years	Referent		Referent		Referent	
3–4 years	1.61 (1.09–2.40)		1.34 (1.02–1.75)		2.95 (1.95–4.47)	
5–6 years	2.30 (1.50–3.54)		1.46 (1.07–2.01)		2.96 (1.46–6.06)	
7–8 years	3.00 (1.85–4.88)		1.59 (1.15–2.20)		4.75 (2.05–11.00)	
>8 years	4.59 (2.72–7.75)		1.76 (1.16–2.67)		9.63 (3.59–25.83)	

Legend Table 5.

All multivariate models were adjusted for age, gender and the parental reported history of the respective (measles/mumps/rubella) infection. In addition, the multivariate measles model was adjusted for age at first measles vaccination and migration background. In all models subjects who had received their first respective (measles/mumps/rubella) vaccination no more than 21 days before were excluded.

* Explained variation [Nagelkerke]: 0.056; n = 11.018.

** Explained variation [Nagelkerke]: 0.057; n = 11.049.

*** Explained variation [Nagelkerke]: 0.075; n = 10.816.

Risk of seronegativity was associated with the number of years that had passed since last vaccination for measles, mumps and rubella. The increase of measles and mumps seronegativity by time since last vaccination was less continuous in one-dose than in two-dose vaccinees. Increase by years since vaccination was continuous for one-dose and two-dose rubella vaccination. Although overall seronegativity was lowest for rubella (see paragraph 3.3) the odds of being seronegative for those who had received the last vaccination more than 4 years ago were highest for rubella regardless of the number of doses.

The odds of being seronegative for mumps antibodies were 3 to 4-fold in one-dose vaccinees regardless of the number of years since the vaccination. In two-dose vaccinees who had received the last dose 3–4 years ago, the odds of being seronegative was 34% higher than in those who had received the last vaccination no more than two years ago and was 76% higher if the vaccination had been given more than 8 years ago.

A clear increase by time since last vaccination was shown for measles vaccination in two-dose vaccinees: The odds of being seronegative was 1.61 (95% CI 1.09–2.40) in those who received the last vaccination 3–4 years ago and increased to 4.59 (95% CI 2.72–7.75) in those who had received the last measles vaccination more than 8 years ago.

## Discussion

### Main results

During 2003 to 2006, concomitant seroprevalence of antibodies to measles, mumps and rubella was below 80% in German children and adolescents, while the vaccination rate was 88.8%. The highest level of seronegativity was seen for antibodies to mumps. Overall, seronegativity was more likely in boys than girls. Differences by gender were most important for rubella and were limited to older age groups. For measles, mumps and rubella seronegativity was highest in young children; seronegativity of measles and rubella antibodies was also higher in foreign born children, in children living in the region of the former West Germany and in children of mothers with a high education level. Most differences could be explained by the respective vaccination coverage which was below the 95%-target value in each age group but was far too low in the youngest and the oldest age group. Risk of seronegativity was highest in unvaccinated children and was lowest in those who had received two doses of the respective vaccine. Seronegativity in one-dose and two-dose vaccinees differed mostly with regard to mumps but even in two-dose vaccinees seronegativity exceeded 5% thus questioning the mumps-vaccine effectiveness. Seronegativity increases as time since last vaccination passes thus indicating significant waning effects for all three components of MMR.

### Measles

Sero-epidemiological findings on measles antibodies has been described and discussed elsewhere [10]. In short, our results showed that seronegativity to measles antibodies in 10 to 17 year-olds seen in our study exceeded the WHO European Region target [13] for measles elimination of <5%. Although Europe had targeted measles elimination by the year 2010, this goal was not met [14] and recently, a new target date for eliminating measles had been set to 2015. Seronegativity was above the overall WHO target level of 5% in children who had received a single dose vaccination, however, the target level was met in those who had received two doses of measles vaccine and the crucial importance of a two-dose vaccination schedule is supported by this population-based German study.

### Mumps

Although seroconversion and/or short-term protective efficacy rate for most vaccine strains of mumps virus was shown to be close to 90% or even >90%, outbreak-studies suggest that the long-term population-based effectiveness of one dose of mumps vaccine islower (60–90%) [6], [15], [16]. One-dose immunization against mumps has been shown to be not sufficient to abrogate mumps virus transmission [17], [18]. In Germany, vaccination coverage against mumps was shown to be 93% and 72% for one dose and two doses of mumps vaccine respectively [19]. Given this vaccination coverage, low seroprevalence of mumps antibodies could be expected.

Our study results support the view that the effectiveness of one dose of mumps vaccine is low. One out of five children who had received one dose mumps vaccine was seronegative for mumps antibodies. Even in children who had received a second mumps vaccination 7.3% were seronegative. As it was the case for measles and rubella, mumps seronegativity was slightly higher in male than in female children. Interestingly, mumps seronegativity was not different between foreign-born and German-born migrants and children without migration background, while differences were observed for measles and rubella. Although the level of seronegativity was about the same for measles, mumps and rubella in foreign-born children, mumps seronegativity was as high in German-born children. Measles- and rubella seronegativity was less frequent in German-born children thus leading to significant differences for measles- and rubella- but not for mumps antibodies. Results indicating complex associations between mumps seronegativity and race/ethnicity and/or a birthplace abroad were also yielded by evaluations of the mumps antibody seroprevalence in the US population [20].

In our study, the overall population-based seronegativity was 15.3% accompanied by a high level of equivocal titres (6.2%). Although no vaccine-induced antibody level has been established as a correlate of protection against mumps disease [21] the number of recent mumps outbreaks may support the vulnerability of the population with a low seroprevalence of mumps antibodies in Germany [22]–[24]. Hypotheses why outbreaks occur in communities with high vaccination coverage involve waning of immunity even after two doses, different neutralization capacity of vaccinees with respect to currently detected mumps virus genotypes and a higher seroprevalence of mumps antibodies that may be needed for meeting the herd immunity threshold [25]. This threshold was previously suggested to be 88–92% [26]. Our study provides population-based, representative sero-epidemiological data for Germany and it was shown that seroprevalence of mumps antibodies is far below the 88–92% needed for mumps control. However, compared to non-representative sero-epidemiological data from 1998, seroprevalence of mumps antibodies improved in all age groups [27]. The remarkable added value of our new analyses was enabled by the study design of the KiGGS survey that allowed combining antibody titres, detailed socio-demographic data and data on the vaccination status of most of the participants on an individual level. On average, only seroprevalence in two-dose vaccinees was sufficient to meet the threshold of 88–92%. However, ELISA seronegativity is not necessarily a synonym for missing immunity, because a protective titre has not been established and the immunity is also cell-mediated. Therefore, seropositivity is not a correlate or not even an ideal surrogate for effectiveness but provides valuable data in addition to outbreak studies. Although the cross-sectional study design of our study does not allow distinguishing between primary and secondary vaccine failure, the multivariate analysis by number of doses and years since last vaccination indicates presence of both factors possibly limiting the vaccine effectiveness: Controlling for time since last vaccination (among others), multivariate analysis showed a 3.7-fold odds of being seronegative in one-dose vaccinees in comparison to two-dose vaccinees. One possible explanation for this observation would be a relevant degree of primary vaccine failure. On the other side, our results would also be in line with seroconversion rates of about 90% after one-dose of mumps vaccine [6] and a rapid decline of mumps antibody level after the first vaccine dose as has been shown by a long-term Finnish study [28], [29],

Studies on the decline in antibody levels after a second dose of mumps vaccination are scarce. Investigation of the association between attack rates and time since last vaccination in outbreak studies yielded conflicting results. Some studies did not find associations [30]–[32] while others showed an association [33]–[35]. In a sero-survey performed in an US-university, seroprevalence did not differ by time since vaccination, however, in this study very few participants were vaccinated less than 6 years before the samples were taken and the main analysis investigated students who had been vaccinated more than 10 years in comparison to those vaccinated no more than 10 years ago [36]. Therefore, a significant decline in mumps antibody levels within 10 years after a second mumps vaccination as seen in the prospective Finnish study [29] and in our population-based study would have remained unnoticed for methodological reasons. Our study results support the presence of secondary vaccine failure as the odds of being seronegative in 2-dose vaccinees increased gradually by years since last vaccination and was increased by 76% if more than 8 years had passed after the second dose of mumps vaccine. Information on mumps antibody level protecting individuals against mumps reinfection would be necessary to assess susceptibility arising from the observed antibody decline. However, our results clearly show, that the high level of seronegativity in one-dose vaccinees (caused either by primary or secondary vaccine failure) is much more important than the lesser decline of antibodies after a second vaccine dose.

After the introduction of routine mumps vaccination it is important to maintain high levels of vaccination coverage. Insufficient vaccination coverage can result in an epidemiological shift in the incidence of mumps to older age groups, potentially leading to serious disease burden [6]. Our data and the experience of recent outbreaks [24], [37]–[40] indicate that an extensive two-dose vaccination coverage in Germany is crucial for mumps control and that increasingly older age groups are likely to contract mumps virus unless catch-up campaigns will increase two-dose vaccination coverage in adolescents.

### Rubella

Our study shows that the overall population-based seroprevalence of rubella antibodies is comparable to measles IgG antibodies. Significant differences by gender have been shown with a high level of seronegativity in boys; differences were highest in adolescents. Vaccination rates in 14 to 17 year old boys have been shown to be lower than in girls [19]. Given the risk of congenital rubella syndrome for future pregnancies, parents often decided to get their daughter (but not the sons) vaccinated against rubella. This approach was even part of the former vaccination schedule and it took some time to overcome this notion. Seronegativity to rubella was more frequent in foreign-born children than in German-born children. In contrast to measles [10] this difference was fully explained by a differing vaccination coverage. Immigrant children are at particular risk of an incomplete immunisation [41]–[46]. In general, data on German vaccination coverage showed lower vaccination coverage for rubella in comparison to measles vaccination. Linking the seroprevalence data to the children's vaccination status showed that seronegativity in one- and two-dose measles-, mumps- and rubella vaccinees was smallest for rubella antibodies. These results are consistent with former studies in Canada and UK [47], [48]. The percentage of seronegative one-dose and two-dose vaccinees is lowest for rubella regardless of the time since last vaccination and support the high immunogenicity of the live attenuated RA 27/3 strain based vaccine [9], [49]. On the other side, our results show that the strengths of association between seronegativity and years since last vaccination is highest for rubella and German population-based seroprevalence data support the view that waning antibody titres may be more obvious for rubella than for measles and mumps. However, rubella antibody waning affects the vaccine effectiveness to a smaller extent than for measles and mumps. These results confirm observations from a smaller study that assessed waning of rubella antibodies by linking vaccination record data and antibody titres on an individual level [50] and a large sero-epidemiological survey that related antibody level to vaccination data from the national vaccination schedules in England and Wales [48].

The main factor associated with seronegativity to measles, mumps and rubella is a missing MMR vaccination. The MMR vaccination rate is lowest in young children leaving the young and thus most vulnerable children without protection against measles-, mumps- and rubella infection. Although the timeliness of vaccination has improved after the availability of combination vaccines [51], vaccination of young children is often delayed and the compliance with the national recommendations needs further improvement [19], [52].

### Strengths and Limitations

Our sero-epidemiological study was conducted in more than 13,000 children and adolescents from the KiGGS survey which were recruited throughout Germany by random population based sampling. Our study thereby overcomes the limitation of former seroprevalence studies that relied on convenience sampling or community based sampling and the study population can be considered representative for German children and adolescents.

Vaccination status was obtained by the detailed vaccination records (vaccination card). By using vaccination cards, validity of the date of vaccination and the administered type of vaccine was high and unaffected by recall problems. This allowed us to identify real vaccination failure rates. However, as others before we cannot be sure that every vaccination had been documented in the provided vaccination card. Although we excluded children from our analyses whose vaccination cards were reported to be incomplete, completeness could not be systematically ensured.

In a study of this size, IgG antibodies must be measured by an automated ELISA procedure. ELISA has a lower sensitivity compared to the plaque reduction neutralization test, which is considered as the gold standard for determining serum antibodies neutralising measles virus [53] and mumps virus [54]–[56], while a similar test for rubella virus has not yet been evaluated [57]. Since immunity for measles, mumps and rubella is cell-mediated, seronegativity is not the equivalent of susceptibility. Another shortcoming of this and similar studies is that no test method can differentiate between immunity after vaccination and natural immunity. To minimize this confounding possibility parents were asked about any clinical history of measles, mumps and rubella by a standardized interview performed by a physician. Previously we estimated the positive and negative predictive values of this parental reported measles history and showed lower NPV in adolescents and lower PPV in young children [10]. Thus, the probabilities of both, undetected wild virus contact and of undocumented vaccination appear to increase with age. These phenomena and a high percentage of clinically inapparent rubella and mumps virus infections may have confounded our results especially in older age groups. An additional limitation for the interpretation of our cross-sectional, seroprevalence study results arises from the fact that primary vaccine failure cannot be distinguished from secondary vaccine failure.

However, on a regular basis German vaccination coverage is only assessed in children prior to school entry. These data are typically incomplete as not all children examined can present their vaccination card. Thus, a nation-wide assessment of titres in correlation with vaccination data substantially improves any evaluation of the rate of protection against measles, mumps and rubella.

## Conclusion

The proportion of MMR seronegative children was highest in the youngest age group and the proportion of an equivocal titre level was highest among adolescents. We identified two main associated factors: a delay of the first MMR vaccination in young children and waning antibody titres in older children. Seronegativity was also higher in older children and adolescents and in foreign-born children for measles and rubella. Seronegativity was highest in unvaccinated children and was above 5% in one-dose vaccinees. In two-dose vaccinees seronegativity was below 5% for measles and rubella but not for mumps. Seronegativity increased by years since last vaccination thus indicating antibody waning for measles, mumps and rubella.

This large population based sero-epidemiological study supplies valuable data with respect to certification of the WHO elimination goals in Germany. Moreover, it supports and confirms once again the crucial importance of a two-dose vaccination schedule to achieve measles and rubella elimination and to control for mumps outbreaks [48], [58], [59]. Timeliness of the first MMR vaccination must be improved in Germany and MMR-catch up vaccination campaigns should focus on adolescents and immigrants.
